# Effect of the Lymphocyte Activation Gene 3 Polymorphism rs951818 on Mortality and Disease Progression in Patients with Sepsis—A Prospective Genetic Association Study

**DOI:** 10.3390/jcm10225302

**Published:** 2021-11-15

**Authors:** Caspar Mewes, Tessa Alexander, Benedikt Büttner, José Hinz, Ayelet Alpert, Aron-F. Popov, Tim Beißbarth, Mladen Tzvetkov, Marian Grade, Michael Quintel, Ingo Bergmann, Ashham Mansur

**Affiliations:** 1 Department of Anesthesiology, University Medical Center, Georg August University, D-37075 Goettingen, Germany; tessa.alexander@med.uni-goettingen.de (T.A.); benedikt.buettner@med.uni-goettingen.de (B.B.); mquintel@med.uni-goettingen.de (M.Q.); ingo.bergmann@med.uni-goettingen.de (I.B.); 2 Center of Anesthesiology and Intensive Care Medicine, University Medical Center Hamburg-Eppendorf, D-20251 Hamburg, Germany; 3 Department of Anesthesiology and Intensive Care Medicine, Klinikum Region Hannover, D-30459 Hannover, Germany; jose.hinz@krh.eu; 4 Department of Immunology, Rapport Faculty of Medicine, Technion-Israeli Institute of Technology, Haifa 31096, Israel; ayelethappy@gmail.com; 5 Department of Cardiovascular and Thoracic Surgery, Helios Clinic Siegburg, D-53721 Siegburg, Germany; aronf.popov@gmail.com; 6 Institute of Medical Bioinformatics, University Medical Center, Georg August University, D-37077 Goettingen, Germany; tim.beissbarth@ams.med.uni-goettingen.de; 7 Department of Pharmacology, University Medical Center, Ernst-Moritz-Arndt-University, D-17487 Greifswald, Germany; mladen.tzvetkov@uni-greifswald.de; 8 Department of General, Visceral and Pediatric Surgery, University Medical Center, Georg August University, D-37075 Goettingen, Germany; marian.grade@med.uni-goettingen.de; 9 Department of Anesthesiology, Asklepios Hospitals Schildautal, D-38723 Seesen, Germany

**Keywords:** LAG-3, lymphocyte-activation gene 3, single nucleotide polymorphism, genetic association study, sepsis, mortality

## Abstract

(1) Background: Sepsis is a leading cause of death and a global public health problem. Accordingly, deciphering the underlying molecular mechanisms of this disease and the determinants of its morbidity and mortality is pivotal. This study examined the effect of the rs951818 SNP of the negative costimulatory lymphocyte-activation gene 3 (LAG-3) on sepsis mortality and disease severity. (2) Methods: 707 consecutive patients with sepsis were prospectively enrolled into the present study from three surgical ICUs at University Medical Center Goettingen. Both 28- and 90-day mortality were analyzed as the primary outcome, while parameters of disease severity served as secondary endpoints. (3) Results: In the Kaplan–Meier analysis LAG-3 rs951818 AA-homozygote patients showed a significantly lower 28-day mortality (17.3%) compared to carriers of the C-allele (23.7%, *p* = 0.0476). In addition, these patients more often received invasive mechanical ventilation (96%) during the course of disease than C-allele carriers (92%, *p* = 0.0466). (4) Conclusions: Genetic profiling of LAG-3 genetic variants alone or in combination with other genetic biomarkers may represent a promising approach for risk stratification of patients with sepsis. Patient-individual therapeutic targeting of immune checkpoints, such as LAG-3, may be a future component of sepsis therapy. Further detailed investigations in clinically relevant sepsis models are necessary.

## 1. Introduction

Sepsis, defined as a life-threatening organ dysfunction caused by a dysregulated host immune response to infection [1], is a global public health problem with an estimated number of 48.9 million annual cases and 11 million recorded sepsis-related deaths in 2017 [2]. It is estimated that the polymorph syndrome of sepsis may be responsible for up to approximately 20% of all global deaths, representing one of the major causes of mortality worldwide [2,3]. With an increasing invasiveness of routine diagnostics and therapeutic interventions and a growing necessity of intensive care patient treatment sepsis has become a pivotal clinical problem for almost all medical disciplines [4]. Furthermore, the current epidemiologic world affair of the Coronavirus disease 2019 pandemic (COVID-19) moved sepsis and inflammation research even more into the focus of medical science [5,6,7].

Due to the fact that the underlying biological and immunological mechanisms causing this disease remain rudimentary explored, it is fundamentally important to investigate the key determinants of sepsis-associated morbidity and mortality [8,9]. It is well known that the clinical phenotype and outcome of sepsis is heterogeneous, affected by various exogenous and endogenous factors. Diagnostic procedures, pathogen characteristics and antibiotic treatment strategies are exogenous factors, for instance [10,11]. Endogenous factors include preexisting conditions and chronic diseases, host immune status, genetic predisposition, age, gender, and many more [12,13,14,15]. The exploration and investigation of the genetic determinants of sepsis as well as inflammatory conditions in general is a popular method, that could be extensively applied in the future [16,17,18,19,20].

Emerging evidence suggests that negative costimulatory immunoregulatory checkpoint proteins such as PD-1 or CTLA-4 play a key role in the regulation of pro- and anti-inflammatory pathways in the clinical syndrome of sepsis [12,21,22,23,24,25]. They are involved in the downregulation of immune-stimulating cell surface molecules, apoptosis of immune cells and T-cell exhaustion leading to immunosuppression and increased susceptibility for secondary nosocomial or opportunistic infections and virus reactivations [26,27,28,29]. Immune cell checkpoints are therefore increasingly being recognized as fundamental contributors to sepsis-related organ dysfunction and mortality [30,31,32]. 

The present study was conducted to evaluate the potential influence of genetic variations in the lymphocyte-activation gene 3 (LAG-3) on outcome and disease progression in a representative cohort of patients with sepsis. LAG-3, also referred to as CD223 (cluster of differentiation 223) is an immunoregulatory cell surface protein expressed on activated T-cells, natural killer cells (NK-cells), B cells, and plasmacytoid dendritic cells [33,34,35,36,37]. It represents an immune checkpoint protein interacting with antigen molecules presented on major histocompatibility class II-receptor (MHCII) on antigen presenting cells (APCs) [37]. Among other functions, LAG-3 is known to negatively regulate T-cell proliferation, activation and homeostasis, similarly to CTLA-4 and PD-1 [38,39]. Previous studies revealed that LAG-3 expression correlates to reduced expansion and increased cell death of effector T-cells [38,39]. Furthermore, LAG-3 has been reported to be involved in the suppressive function of regulatory T-cells; LAG-3 expression plays a role in mediating suppression by natural CD4-positive, CD25-positive (CD4+/CD25+) regulatory T-cells, and additionally in the regulation of homeostatic lymphocyte expansion by natural regulatory T-cells [40].

The LAG-3 rs951818 single nucleotide polymorphism (SNP) is located in a non-coding region downstream of LAG-3 and may have a regulatory function on the expression of LAG-3 [40]. Genetic variants at this position have been previously identified as a potential risk factor to the chronic inflammatory central nervous system (CNS) disease of multiple sclerosis (MS) [41,42]. Furthermore, the CC-genotype of the rs951818 SNP was found to be significantly higher in female Parkinson’s disease (PD) patients than in controls [40]. 

Based on previous investigations we hypothesized that genetic variants at the rs951818 position could associate to altered disease severity and outcome of patients with sepsis.

## 2. Materials and Methods

### 2.1. Patients

For the purpose of the present study, we prospectively enrolled 707 consecutive patients with clinically defined sepsis from three surgical intensive care units (ICUs) at the University Medical Center Goettingen, Germany, since 2012. Our cohort of sepsis patients, or significant proportions of it, were previously studied in other published investigations for other clinical and experimental research questions by our study group [14,15,16,21,24,25,43]. Patient screening for sepsis and study recruitment was performed by study physicians on a daily basis using the currently valid international sepsis definitions and guidelines [1,44,45,46]. Eligible patients were added to the study data base and followed up for a maximum of 28 days unless they were previously discharged from ICU or deceased. No patient was lost to follow-up. The 90-day mortality was manually collected by personal telephone follow-up or official written request at the local registry. The following previously described study exclusion criteria were applied [14,24,25]:

(I) Patient under immunosuppressive therapy or cancer-related chemotherapy;
(II) Myocardial infarction within six weeks before study enrolment;
(III) Chronic infection with human immunodeficiency virus (HIV);
(IV) Congestive heart failure New York Heart Association (NYHA) level IV;
(V) End-stage incurable disease with a reduced probability of surviving the following 28 days;
(VI) Pregnancy or breastfeeding;
(VII) Patient aged below 18 years;
(VIII) “Do Not Resuscitate” (DNR) or “Do Not Treat” (DNT) order;
(IX) Patient in persistent vegetative stage (apallic syndrome);
(X) Patient participation in interventional studies;
(XI) Familial relationship to a member of the study team.

### 2.2. Data Collection

Predefined patient baseline data and clinical parameters were recorded daily during the 28-day observation period using clinical report forms (CRFs) and the GENOSEP database of the Department of Anesthesiology and Intensive Care Medicine of the University Medical Center Goettingen, Germany. 

Patient baseline characteristics included basic conditions such as age, gender, body mass index (BMI), the baseline disease severity described by day one Sequential Organ Failure Assessment (SOFA) [1] and Acute Physiology and Chronic Health Evaluation (APACHE-II)-scores [47], Procalcitonin (PCT) measure, and the use of organ support (mechanical ventilation, use of vasopressors, and renal replacement therapy). Furthermore, common comorbidities, preexisting medication, recent surgical history, and primary site of infection were recorded.

Disease severity analysis involved daily recorded SOFA scores, the manifestation of clinically defined septic shock and the number of days the patient was in septic shock. Further parameters included inflammatory measures including leukocyte count, serum C-reactive protein levels (CRP), PCT, and the presence of fever. Organ-specific values of respiration, coagulation, liver and renal function, the central nervous system (CNS), and cardiovascular system were furthermore recorded and evaluated. 

All patient data were generated from the electronic patient record system (IntelliSpace Critical Care and Anesthesia (ICCA), Phillips Healthcare, Andover, MA, USA). 

### 2.3. Genotyping

DNA extraction and SNP genotyping was performed according to the manufacturer’s instructions in the laboratories and under the supervision of the Department of Clinical Pharmacology, University Medical Center Goettingen, Germany. 

Whole blood samples were drawn from all study subjects within 72 h after sepsis onset. The extraction of genomic DNA was performed using either the QIAmp^®^ DNA Blood Kit in QIAcube^®^, the EZ1^®^ DNA Blood Kit in BioRobot EZ1^®^ or the AllPrep DNA Mini Kit (all from Qiagen, Hilden, Germany), as previously described [21,24,43]. Quantity and quality of the extracted DNA were tested by spectrophotometric measurement.

The LAG-3 rs951818 was genotyped in all samples through TaqMan polymerase chain reaction (PCR) using the appropriate predesigned TaqMan^®^ SNP Genotyping Assay C___8921385_10 (Thermo Fisher Scientific, Waltham, MA, USA) and a 7900HT Fast-Real-Time PCR System (Life Technologies, Darmstadt, Germany) as well as 7900HT Fast-Real-Time PCR System software (SDS v2.4.1 for Windows 7, Applied Biosystems, Foster City, CA, USA). Over 20% of the samples were genotyped in duplicate to increase reliability.

### 2.4. Statistical Analysis

Statistical analyses were conducted using STATISTICA 13 software (version 13.5.0.17, StatSoft, Tulsa, OK, USA). *p*-values < 0.05 were considered statistically significant. Associations between categorical variables were analyzed using either Pearson’s chi-square test or two-sided Fisher’s exact test, as appropriate. Discrete variables are presented as absolute numbers or percentages. Continuous variables were tested by Mann–Whitney U test and expressed as mean values ± standard deviations or as median and interquartile ranges (IQRs), where applicable. Kaplan–Meier survival analyses involved the log rank test, whereas adjusted hazard ratios (HR) were calculated using multivariate Cox regression analysis. 

Accordance of SNP genotypic frequencies with Hardy–Weinberg equilibrium was tested by the chi-square test.

## 3. Results

### 3.1. Allele Distribution

We collected data from a total of 707 prospectively enrolled patients with clinically defined sepsis. DNA was successfully extracted, and all study participants were genotyped for the LAG-3 rs951818 SNP according to the above-mentioned experimental protocols. At the investigated LAG-3 rs951818 SNP position, the population’s observed allele distribution was 277:338:92 (AA:AC:CC). Hence, we calculated a minor allele frequency (MAF) of 0.361, which nearly equaled the expected MAF of the European HapMap reference population of 0.356 [48]. The observed allele frequencies of the study population were in Hardy–Weinberg equilibrium (χ^2^ test *p* = 0.494).

For all of the following analyses, AC heterozygote patients (*n* = 338) were pooled with CC homozygotes (*n* = 92; combined *n* = 430) and compared to AA homozygotes (*n* = 277). 

### 3.2. Baseline Characteristics

At the time of enrollment, this study’s participants were on average 63 ± 15 years old, and 65% of them were male (Table 1). Average SOFA- and APACHE II-scores of 10 ± 4 and 22 ± 7 at baseline and a substantial need for organ support (70% vasopressor need, 86% mechanical ventilation and 10% renal replacement therapy) indicate the population’s critical state of health and need for intensive care treatment. The majority of patients underwent emergency (52%) or elective surgery (27%) and presented the lung (63%) or abdomen (19%) as the primary site of infection. Common comorbidities were arterial hypertension (57%), chronic obstructive pulmonary disease (COPD, 15%), and a history of any type of cancer (14%), while preexisting medication commonly included antihypertensive agents (37% beta-blockers, 29% ACE inhibitors), diuretics (34%), and anticoagulation in the previous six months (26%). 

Table 1 shows that there were no statistically significant differences in patient baseline characteristics between the two groups of AC/CC-genotypes and AA-homozygotes. 

### 3.3. Kaplan–Meier Survival Analysis

**Figure 1:**
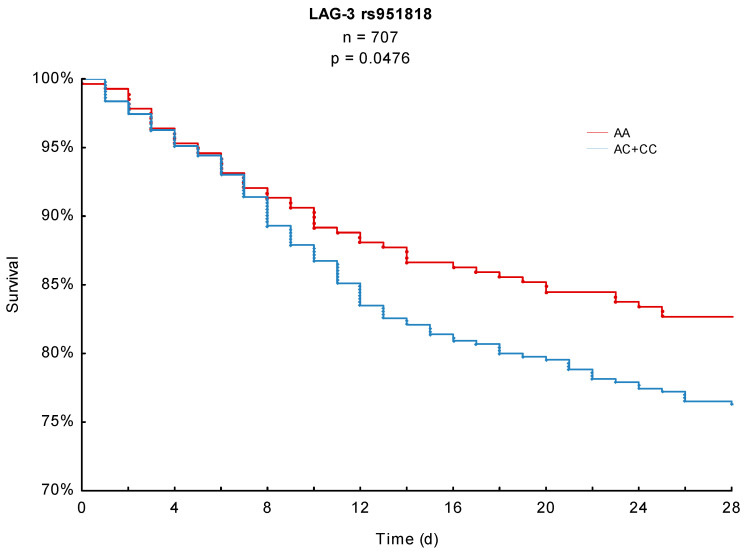
Kaplan–Meier 28-day survival analysis with respect to the LAG-3 rs951818 SNP.

**Figure 2:**
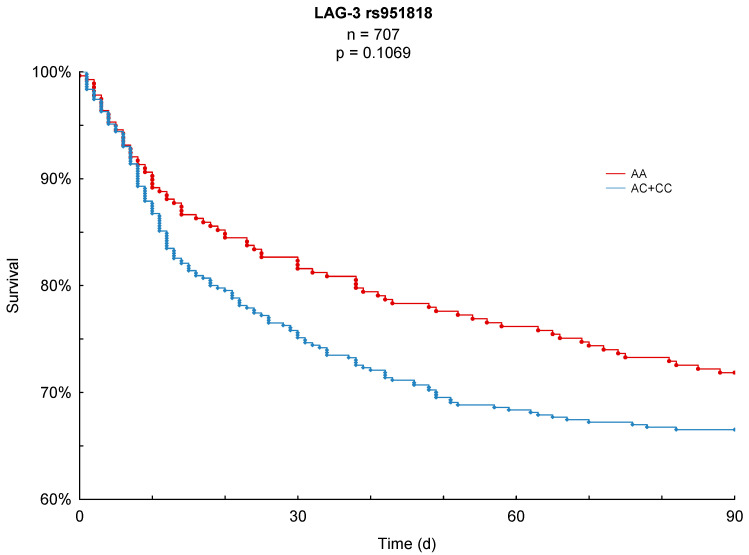
Kaplan–Meier 90-day survival analysis with respect to the LAG-3 rs951818 SNP.

Both 28- and 90-day mortality served as the primary endpoints of this study. In the conducted Kaplan–Meier survival analyses, LAG-3 rs951818 AA-homozygotes showed a significantly lower 28-day mortality (17.3%) compared to patients with the AC or CC genotype (23.7%, *p* = 0.0476, Figure 1). For the 90-day observation period AA-homozygotes also showed a lower mortality (28.2%) in comparison to AC/CC-genotypes (33.5%) in the study cohort; this finding, however, did not reach statistical significance (*p* = 0.1069, Figure 2). 

### 3.4. Disease Severity

The performed analysis of disease severity involved common measures of inflammation and sepsis severity as well as organ-specific parameters. 

Our analysis revealed that AA-homozygote patients received significantly more invasive mechanical ventilation (96% of the AA-homozygotes) compared to carriers of the AC or CC genotype (92%, *p* = 0.0466, Table 2). 

While some of the other observed disease severity values showed differences between the two compared groups, none of them were statistically significant.

### 3.5. Multivariate Cox Regression Analysis

In order to eliminate the effect of potential confounders, we included age, gender, BMI, SOFA-score on sepsis onset, as well as APACHE II-score in the multivariate Cox regression analysis. As we did not find significant differences in the patient baseline characteristics no other parameters were added to this analysis. 

The multivariate model revealed, that a higher age and higher SOFA-score on sepsis onset have a significantly negative effect on 28- and 90-day mortality, whereas a higher BMI seemed to have beneficial effects on 28-day mortality (Table 3). 

However, the LAG-3 rs951818 AA-genotype did not remain to have a significant effect on 28- or 90-day mortality (*p* = 0.0981 and *p* = 0.1755, respectively, Table 3) after adjustment for potential confounders in this model. 

## 4. Discussion

To the best of our knowledge, we present the first prospective investigation of the association between the LAG-3 rs951818 SNP and the survival of patients with sepsis. As a main result, our study revealed that LAG-3 rs951818 AA-homozygote patients had a significantly lower 28-day mortality in sepsis compared to carriers of the C-allele (17.3% vs. 23.7%, *p* = 0.0476). Furthermore, carriers of the AA-genotype at this position also presented a better long-term survival in the observation period of 90 days (28.2%) compared to carriers of the C-allele (33.5%); this finding was, however, not statistically significant (*p* = 0.1069).

As a secondary endpoint of this investigation, we observed that AA-homozygote patients received significantly more mechanical ventilation as organ-support during the course of disease compared to C-allele carriers (96% vs. 92%, *p* = 0.0466). 

These findings further support previous investigations demonstrating that specific cell surface inhibitory immune checkpoint receptors and ligands such as PD-1, PD-L1, CTLA-4, TIM-3, and LAG-3 play a critical role in maintaining immune homeostasis in sepsis [49]. Their function to limit excess inflammation physiologically mediates the balance between host immune competency and immunosuppression. A variety of genetic variants in immune checkpoint genes as well as pattern recognition receptor, cytokine, and other immune-related genes have been reported to significantly correlate to the clinical course or mortality of sepsis in the past years [15,16,21,24,25,43,49,50]. 

Upregulated levels of the inhibitory immune checkpoint molecule LAG-3 were previously observed in a cecal ligation puncture (CLP) model of murine sepsis [51]. In their study Lou et al. showed increased LAG-3 expressions on CD4- and CD8-positive T cells as well as on B cells, dendritic cells and regulatory T cells (Treg) [51]. Furthermore, treatment with anti-LAG-3 antibody improved the ability to clear primary infections, reduced the incidence of secondary nosocomial infections caused by opportunistic pathogens, and thereby improved survival after sepsis [51]. Likewise, a recent study by Niu et al. showed, that the co-expression of LAG-3 and PD-1 synergistically inhibited CD4- and CD8- positive T cells, which correlated with a higher mortality and hospital length of stay [52]. 

The exact function of the studied genetic variation at the LAG-3 rs951818 position is not fully understood, but it may be assumed that it has a regulatory function on LAG-3 expression and it was previously shown to correlate with the incidence of MS and PD [40,41,42]. We suppose that altered expression levels of LAG-3 and/or impaired protein function are the rationale behind our finding of advantages of the LAG-3 rs951818 AA-genotype in sepsis survival. As far as the multivariate regression analysis did not confirm this genotype’s independent predictive or prognostic value, it must be considered that other factors co-affect the observed improved survival. 

This study is particularly strong as it involved a large, clearly defined, homogenous, and prospective cohort of patients with sepsis. However, it has some limitations. The investigation was performed in a monocentric study design and should be further validated in independent other sepsis cohorts. Due to the lack of significance in the performed multivariate model, the LAG-3 rs951818 SNP cannot be considered as an unlimited predictive variable for sepsis survival, which does however conform to the assumption, that the course of disease in sepsis is multifactorially affected and certainly polygenetic. Furthermore, this study only involved patients from surgical ICUs, so that observations may not be representative for other ICUs (e.g., medical ICU). Moreover, future investigations should correlate the LAG-3 rs951818 polymorphism to additional hematologic parameters including differential blood counts, neutrophil–lymphocyte-ratio, cytokines, or LAG-3 expression levels in order to further and more directly reflect the association between LAG-3 and the humoral and cellular host immune reaction. 

The authors believe that further investigation of the LAG-3 rs951818 function and the underlying biological mechanisms holds significant potential for a better understanding of the multi-faceted syndrome of sepsis. Patient risk stratification according to genetic profiles and individualized immunotherapies with immune cell checkpoint inhibitors, whether anti-LAG-3 or a combination with others, are surely a future component of sepsis therapy.

## Figures and Tables

**Table 1:** Baseline characteristics regarding LAG-3 rs951818 SNP.

Characteristics	All (*n* = 707)	AC/CC (*n* = 430)	AA (*n* = 277)	*p*-Value
Basic conditions				
Age (years)	63 ± 15	64 ± 15	63 ± 15	0.1979
Male gender (%)	65	64	66	0.5844
Body Mass Index (BMI) (kg/m²)	28 ± 7	28 ± 7	28 ± 6	0.8869
Severity on Sepsis Onset (Day 1)				
SOFA-score	10 ± 4	10 ± 4	10 ± 4	0.4833
APACHE II-score	22 ± 7	22 ± 7	22 ± 7	0.5983
Procalcitonin (ng/dL)	1.3 (0.5–4.8) (*n* = 343)	1.4 (0.5–4.8) (*n* = 218)	1.2 (0.4–5.1) (*n* = 125)	0.6158
Use of vasopressor (%)	70	72	67	0.1512
Mechanical ventilation (%)	86	85	89	0.1370
Renal replacement therapy (%)	10	9	11	0.5067
Comorbidities (%)				
Arterial hypertension	54	54	53	0.6560
COPD	15	16	14	0.5299
Bronchial asthma	3	3	2	0.6068
Renal dysfunction	10	10	10	0.7580
Non-insulin-dependent diabetes mellitus (NIDDM)	9	8	10	0.3949
Insulin-dependent diabetes mellitus (IDDM)	10	11	10	0.6852
Chronic liver disease	6	6	5	0.6352
History of myocardial infarction	6	6	5	0.9247
History of stroke	6	6	5	0.3729
History of cancer	14	12	16	0.1508
Medication on Sepsis Onset (Day 1) (%)				
Statins	23	23	24	0.7214
Beta-blocker	37	40	33	0.0631
ACE inhibitor	29	30	29	0.7219
Bronchodilator	10	11	9	0.3149
Diuretic	34	35	31	0.2632
Anticoagulation in last 6 months	26	27	24	0.4479
Recent surgical history (%)				
Elective surgery	27	27	28	
Emergency surgery	52	50	54	0.2535
No surgery	21	23	18	
Site of infection (%)				
Lung	63	62	64	
Abdomen	19	19	18	
Bone or soft tissue	4	4	3	
Surgical wound	2	2	2	0.8131
Urogenital	2	2	3	
Primary bacteremia	6	7	5	
Other	5	4	6	

Categorical variables are presented as absolute numbers or percentages; continuous variables are presented as the mean values ± the standard deviations or as median and interquartile ranges (IQRs).

**Table 2:** Clinical parameters and disease severity analysis regarding LAG-3 rs951818 SNP.

Characteristics	All (*n* = 707)	AC/CC (*n* = 430)	AA (*n* = 277)	*p*-Value
Sepsis severity				
SOFA-score	7.2 ± 3.7	7.3 ± 3.6	7.1 ± 3.8	0.2891
Septic shock (%)	50	53	46	0.0876
Days in septic shock	1 (0–2)	1 (0–2)	0 (0–2)	0.1912
Inflammatory values				
Leucocytes (1000/µL)	13.3 ± 5.1	13.5 ± 5.2	12.9 ± 4.9	0.0991
C-reactive protein (mg/L)	149.9 ± 85.9	154.0 ± 91.6	143.4 ± 76.0	0.5112
Procalcitonin (ng/dL)	1 (0.3–3.4) (*n* = 628)	1 (0.4–3.3) (*n* = 380)	0.8 (0.3–3.5) (*n* = 248)	0.2249
Fever (%)	88	86	90	0.1584
Respiratory values				
SOFA respiratory subscore	2.0 ± 0.8	2.0 ± 0.8	2.0 ± 0.8	0.8359
Mechanical ventilation (%)	94	92	96	0.0466
Ventilated days/observation (%)	68 ± 32	68 ± 33	68 ± 30	0.3147
Coagulation				
SOFA coagulation subscore	0.4 ± 0.6	0.4 ± 0.6	0.4 ± 0.7	0.6427
Thrombocytes (1000/µL)	291 ± 150	287 ± 144	297 ± 160	0.4988
Liver values				
SOFA hepatic subscore	0 (0–0.4)	0 (0–0.5)	0 (0–0.4)	0.9448
Bilirubin (mg/dL)	0.6 (0.4–1.1)	0.6 (0.4–1.1)	0.6 (0.4–1.1)	0.8242
AST (GOT) (IU/L)	57 (35–112) (*n* = 462)	58 (35–119) (*n* = 288)	54 (32–106) (*n* = 174)	0.4947
ALT (GPT) (IU/L)	46 (23–92) (*n* = 683)	46 (22–92) (*n* = 417)	46 (25–91) (*n* = 266)	0.6155
Cardiovascular values				
SOFA cardiovascular subscore	1.6 ± 1.0	1.7 ± 1.0	1.5 ± 1.0	0.0740
Vasopressor treatment (%)	81	83	78	0.1120
Vasopressor days/observation (%)	36 ± 31	38 ± 31	34 ± 32	0.1117
Central nervous system				
SOFA CNS subscore	2.1 ± 1.1	2.1 ± 1.1	2.0 ± 1.1	0.3201
Glasgow Coma Score (GCS)	9.8 ± 3.2	9.7 ± 3.3	9.9 ± 3.2	0.3606
Renal values				
SOFA renal subscore	0.2 (0–1.2)	0.2 (0–1.1)	0.2 (0–1.3)	0.3936
Creatinine (mg/dL)	1.2 ± 0.9	1.3 ± 1.0	1.2 ± 0.9	0.2136
Urine output (mL/d)	2893 ± 1339	2872 ± 1333	2924 ± 1350	0.4172
Urine output (mL/kg/d)	1.5 ± 0.8	1.5 ± 0.8	1.5 ± 0.8	0.6968
Renal replacement therapy (%)	23	23	21	0.4972
Dialysis days/observation (%)	0 (0–0)	0 (0–0)	0 (0–0)	0.6725

Categorical variables are presented as absolute numbers or percentages; continuous variables are presented as the mean values ± the standard deviations or as median and interquartile ranges (IQRs).

**Table 3:** Multivariate Cox regression analysis with regard to 28-day and 90-day mortality.

Variable	Hazard Ratio	95% CI	*p*-Value
28-day mortality			
Age	1.03	1.02–1.04	<0.001
Male gender	1.15	0.82–1.63	0.4165
Body Mass Index	0.96	0.93–0.99	0.0157
SOFA-score on sepsis onset	1.09	1.04–1.15	0.001
APACHE II-score	1.03	1–1.06	0.0609
LAG-3 rs951818 AA-genotype	0.75	0.53–1.06	0.0981
90-day mortality			
Age	1.03	1.02–1.04	<0.001
Male gender	1.01	0.77–1.34	0.93
Body Mass Index	0.99	0.96–1.01	0.1781
SOFA-score on sepsis onset	1.08	1.03–1.12	0.0006
APACHE II-score	1.03	1–1.06	0.0273
LAG-3 rs951818 AA-genotype	0.83	0.63–1.09	0.1755

## Data Availability

The datasets generated and/or analyzed during the current study are available from the corresponding author on reasonable request.

## Abbreviations

ACE	Angiotensin converting enzyme
ALT	Alanine transaminase
APACHE II	Acute Physiology and Chronic Health Evaluation II
APC	Antigen-presenting cell
AST	Aspartate transaminase
BMI	Body mass index
CD223	Cluster of differentiation 223
CI	Confidence interval
CLP	Cecal ligation puncture
CNS	Central nervous system
COPD	Chronic obstructive pulmonary disease
COVID-19	Coronavirus 2019
CRP	C-reactive protein
CTLA-4	Cytotoxic T-lymphocyte-associated protein 4
GCS	Glasgow Coma Score
ICU	Intensive care unit
IDDM	Insulin-dependent Diabetes mellitus
IQR	Interquartile range
LAG-3	Lymphocyte-activation gene 3
MHCII	Major histocompatibility class II
MS	Multiple sclerosis
NIDDM	Non-insulin-dependent Diabetes mellitus
NK-cell	Natural killer cells
NYHA	New York Heart Association
PCR	Polymerase chain reaction
PCT	Procalcitonin
PD	Parkinson’s disease
PD-1	Programmed cell death protein 1
SD	Standard deviation
SNP	Single nucleotide polymorphism
SOFA	Sequential Organ Failure Assessment
Treg	Regulatory T cell
